# Hyperactive Delirium Among Home Hospice Patients: Improving Death Literacy Through Community-Based Research

**DOI:** 10.1093/geroni/igaf122.2099

**Published:** 2025-12-31

**Authors:** Jordan Whittier, Carol Weisse

**Affiliations:** Union College, Strafford, New Hampshire, United States; Union College, Schenectady, New York, United States

## Abstract

Hyperactive delirium (HD), characterized by restlessness, agitation, hallucinations, and delusions, can be distressing to patients and their caregivers. Symptoms have been reported to increase as death approaches in palliative care and inpatient settings, but less is known about HD occurring in home hospice settings where care is often provided by informal caregivers. This study was conducted through an experiential learning program, that included serving as a caregiver to hospice patients experiencing HD. Using a mixed methods approach, a data registry containing caregiver narratives of 101 hospice patients was queried to gain a deeper understanding of the frequency and severity of HD occurring during patients’ last week of life. Severity was assessed using a modified version of 2 delirium scales (CRS; NU-DESC), coding symptoms as mild (1) or severe (2) based on references to aggression, distress, and safety threats and accompanying descriptors (e.g. extremely) about intensity/duration. Results indicated 23.7% of patients (N = 24) experienced HD, with behavioral symptoms documented more than cognitive ones. Severe symptoms were reported in 95.8% of patients, and incidents peaked four days before death with the most severe symptoms occurring five days before death. Content analysis highlights themes of caregiver challenges, barriers to quality care, and situations placing caregivers at risk for burnout. Results indicate that HD can be frequent and severe during home hospice care and underscore the need for additional support at the end-of-life. The author will share firsthand experience managing HD and discuss the interplay between research and practice.

